# Computing Skin Cutaneous Melanoma Outcome From the HLA-Alleles and Clinical Characteristics

**DOI:** 10.3389/fgene.2020.00221

**Published:** 2020-03-26

**Authors:** Anjali Dhall, Sumeet Patiyal, Harpreet Kaur, Sherry Bhalla, Chakit Arora, Gajendra P. S. Raghava

**Affiliations:** ^1^Department of Computational Biology, Indraprastha Institute of Information Technology, New Delhi, India; ^2^Bioinformatics Centre, CSIR-Institute of Microbial Technology, Chandigarh, India

**Keywords:** cutaneous melanoma, survival analysis, HLA, superalleles, Hazard ratio, regression, machine learning, prognosis

## Abstract

Human leukocyte antigen (HLA) are essential components of the immune system that stimulate immune cells to provide protection and defense against cancer. Thousands of HLA alleles have been reported in the literature, but only a specific set of HLA alleles are present in an individual. The capability of the immune system to recognize cancer-associated mutations depends on the presence of a particular set of alleles, which elicit an immune response to fight against cancer. Therefore, the occurrence of specific HLA alleles affects the survival outcome of cancer patients. In the current study, prediction models were developed, using 401 cutaneous melanoma patients, to predict the overall survival (OS) of patients using their clinical data and HLA alleles. We observed that the presence of certain favorable superalleles like HLA-B^∗^55 (HR = 0.15, 95% CI 0.034–0.67), HLA-A^∗^01 (HR = 0.5, 95% CI 0.3–0.8), is responsible for the improved OS. In contrast, the presence of certain unfavorable superalleles such as HLA-B^∗^50 (HR = 2.76, 95% CI 1.284–5.941), HLA-DRB1^∗^12 (HR = 3.44, 95% CI 1.64–7.2) is responsible for the poor survival. We developed prediction models using key 14 HLA superalleles, demographic, and clinical characteristics for predicting high-risk cutaneous melanoma patients and achieved HR = 4.52 (95% CI 3.088–6.609, *p*-value = 8.01E-15). Eventually, we also provide a web-based service to the community for predicting the risk status in cutaneous melanoma patients (https://webs.iiitd.edu.in/raghava/skcmhrp/).

## Introduction

The HLA complex is the highly polymorphic genetic region located on chromosome 6, precisely in the 6p21.3 region (Beck and Trowsdale, 2000; Choo, 2007). Major histocompatibility complex (MHC) encodes more than 200 immune-related genes, from which approximately 40 genes are associated with the development of leukocyte antigen, i.e., class I and class II HLA genes (Bonamigo et al., 2012). Class I and II regions are categorized into classical and non-classical, where, classical HLA-class I comprises of HLA-A, HLA-B, and HLA-C, and class II HLA gene loci are HLA-DR, HLA-DP, and HLA-DQ (Shiina et al., 2009). Out of which, class I genes encode proteins which present endogenous antigen to CD8 + T lymphocytes, while, class II genes encode proteins which present exogenous antigens to CD4 + T cells (Watts, 2004; Traherne, 2008; Cruz-Tapias et al., 2013). The class I complex is generally located on all nucleated cell surfaces, and class II genes are expressed on the specific antigen-presenting cells (APCs), B lymphocytes and activated T cells (Choo, 2007). By the cross-presentation process, certain APCs present exogenous antigens on HLA class I molecules for the activation of cytotoxic CD8 + T cells responses (Bevan, 2006; Joffre et al., 2012).

HLA molecules play a significant role in the induction and regulation of immune responses. The role of HLA class I molecules has been implied in tumor resistance to apoptosis (Sabapathy and Nam, 2008). Recent findings suggest that the altered expression of HLA molecules is associated with metastatic progression and poor prognosis in the tumor (Aptsiauri et al., 2007; Mendez et al., 2009; Johansen et al., 2016). The modification of surface molecules, lack of co-stimulatory molecules, production of immunosuppressive cytokines, and alterations in HLA molecules are some of the primary escape mechanisms used by tumor cells to evade the immune response (Garrido et al., 2010), which can directly distress the survival of an individual. Previous studies reveal that cutaneous melanoma is one of the most threatening and fatal form of skin cancer and scrutinized multi-omics signatures for the progression of malignancy (Li et al., 2015; Ossio et al., 2017; Bhalla et al., 2019). Further, in the past, it has been shown that if melanoma is detected at an early stage, the OS rate is 95%; but, once it is metastasized (lesion thickness > 4 mm); they are tough to cure, and the survival rate is reduced to less than 50% (Büttner et al., 1995; Bristow et al., 2010). Therefore, tumor staging is crucial to provide fundamental prognostic information to clinicians. To this end, the American Joint Committee on Cancer (AJCC), and the Melanoma Staging Committee, provides information related to Tumor-Nodes-Metastasis (TNM) classification and tumor stage grouping (Gershenwald et al., 2017). Primary tumors (stage I and II), are categorized into T1, T2, T3, and T4 with a corresponding tumor thickness such as ≥1.00 mm, 1.01 – 2.0 mm, 2.01 – 4.0 mm and >4.0 mm, respectively. Regional Lymph Nodes (stage III) are classified into N0, N1, N2, and N3, which represent the number of metastatic tumor nodes (0, 1, 2–3, 4+), respectively. Distant metastasis (stage IV) is divided into four categories M0 (No distant metastases), M1a (metastasis to distant skin, subcutaneous tissues, and/or lymph nodes), M1b (metastasis to the lungs), and M1c (metastasis to any non-pulmonary visceral site) (Gershenwald et al., 1998; Dickson and Gershenwald, 2011). Earlier, it has been observed that melanoma tumor cells escape the immune checkpoints and proliferate at a higher rate than normal tissue cells (Khair et al., 2019). Further, it is categorized as an immunogenic tumor as its lesions have been found to have signatures of several immune escape mechanisms such as the downregulated expression of HLA molecules, secretion of cytokines like IL-10, and loss of tumor-specific antigens (Nestle et al., 1997). For instance, the downregulation of class I antigen have been associated with the poor prognosis and inadequate treatment in melanoma cases (Sun and Schuchter, 2001; Cabrera et al., 2007; Carretero et al., 2008). Moreover, recent studies demonstrate the importance of HLA alleles in the prognosis of melanoma. For example, the loss of heterozygosity in HLA class I allele (HLA-B^∗^15:01) has been shown to be related with poor survival outcome. In addition, HLA-C alleles and the HLA-B44 supertype has been shown to enhance OS (Campillo et al., 2006; Gogas et al., 2010; Chowell et al., 2018), thereby showing that these molecules could be considered prognostic markers for melanoma. Thus, it is vital to ascertain the role of class I and II antigen in the survival of melanoma patients. With the knowledge of accurate HLA typing, one can design immunotherapy-based prognostic biomarkers and personalized vaccines against cancer.

In the current study, we have attempted to understand the role of HLA (class I and II) alleles and superalleles in the survival of cutaneous melanoma patients using The Cancer Genome Atlas (TCGA-SKCM) dataset. Here, first, we have performed the HLA typing of patients for class I and II alleles, followed by their assignment to superallele (i.e., low-resolution HLA alleles) groups. Subsequently, we categorized the HLA superalleles into survival favorable and unfavorable groups based on the significant impact of their presence on the survival of patients. Further, we have developed survival prediction models employing key HLA superalleles, and demographic and clinical features of patients by using different machine learning techniques. In service of the scientific community, we have also developed a webserver “SKCMhrp” to predict low-risk and high-risk patient groups based on HLA superalleles, and clinical and demographic features.

## Materials and Methods

### Study Design and Dataset Collection

**FIGURE 1:**
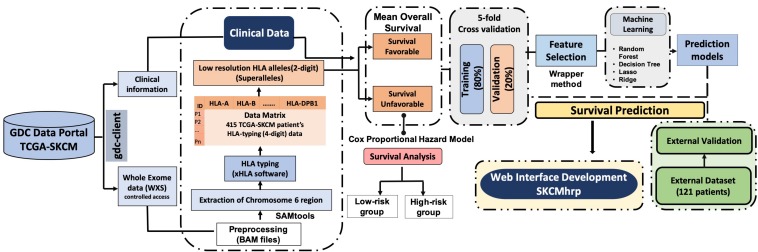
Pipeline representing the workflow of the study.

The complete pipeline of the study is illustrated in Figure 1. The description of each step is given below.

### Dataset Collection

#### Collection of Whole-Exome Dataset

We obtained the TCGA-SKCM controlled access dataset from the Genome Data Commons (GDC) data portal. Specifically, the whole-exome sequencing (WXS) BAM files of individual melanoma patients were downloaded [under the approval of dbGap (Project No. 17674)] according to the GDC protocols (Grossman et al., 2016) with the help of an in-house high-performance computing (HPC) facility and scripts. Clinical information for 470 patients was also obtained, that included age, gender, stage, tumor status, treatment status, Breslow depth, vital status, OS, etc. using TCGA assembler 2 (Zhu et al., 2014; Liu et al., 2018). We were able to extract the HLA typing information for 415 out of 470 TCGA-SKCM patients only, after removing irrelevant errors in the BAM files. Out of 415 samples, 14 patients lacked OS information. In summary, we used 401 cutaneous melanoma patients for which complete survival information with exome sequencing data was available. Clinical information like the type of melanoma, tumor stage, tumor site, Breslow depth, treatment etc., of the patients is shown in Supplementary Table S1.

#### Dataset for Prediction Models

We used the TCGA-SKCM dataset to train our prediction models and assessed the performance of our models using a particular set of features, which included HLA alleles and clinical characteristics. Eventually, the performance was evaluated on the external dataset. For the external validation dataset, we collected data from 121 cutaneous melanoma patients from various studies (Snyder et al., 2014; Van Allen et al., 2015; Hugo et al., 2016; Riaz et al., 2017), which incorporated 145 unique class I and II HLA alleles with two demographics (age and gender) and one clinical feature (tumor stage). We trained our machine learning model on the TCGA-SKCM dataset and evaluated it on the external dataset with a similar set of features.

### HLA Typing

After downloading the whole exome BAM files of cutaneous patients from TCGA, chromosome 6 was extracted from these BAM files using the SAMtools package (Li et al., 2009). Subsequently, we used xHLA software (Xie et al., 2017) for HLA typing from the chromosome 6 region. In this study, four-digit HLA typing was performed for each patient for the assignment of both class I (-A, -B, -C) and class II (-DP, -DQ, -DR) HLA alleles, which are represented in Supplementary Table S2.

### HLA Superallele

**FIGURE 2:**
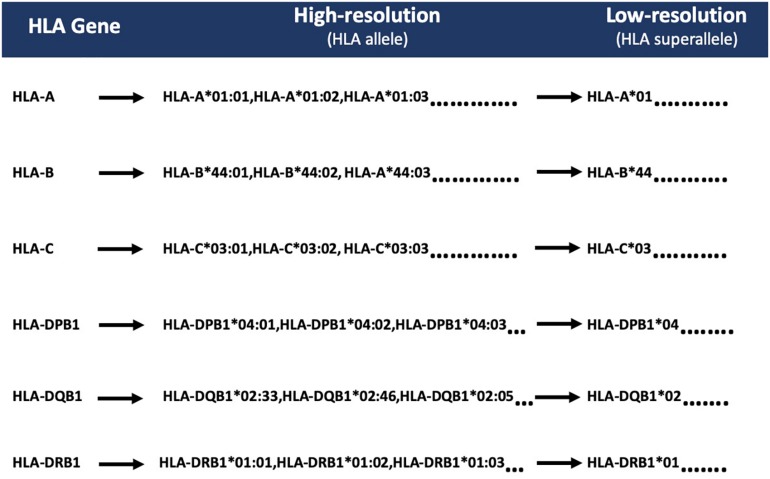
Representation of HLA superalleles on the basis of common HLA gene (-A, -B, -C, -DPB1, -DQB1, -DRB1) and field1 (F1).

According to IMGT/HLA nomenclature, each HLA-allele is assigned to a unique name, followed by the asterisk (^∗^) and separated by colons (Marsh, 2003; Robinson et al., 2016). The first two-digits represent an allele group (field1) (Listgarten et al., 2008); the third and fourth digit corresponds to the specific HLA protein (field2). Due to low-frequency distribution of high-resolution HLA alleles among patients, we combined the HLA alleles on the basis of field1 [which correspond to the historical serological antigen group (or allele family)] (Giannopoulos and Kriebardis, 2017) to form low-resolution HLA alleles. In this study, we used the term “superallele” for the first time for low-resolution HLA alleles, in which we assigned a high resolution (i.e., four-digit typing) to low-resolution (i.e., two-digit typing) HLA allele. For example, HLA-A^∗^01:01/A^∗^01:02/A^∗^01:03 alleles are assigned to the HLA-A^∗^01 superallele. The complete representation of the superallele is shown in Figure 2, and Supplementary Table S2.

### Categorization of HLA Superalleles

Here, we categorized HLA superalleles into favorable and unfavorable groups based on the impact of their presence on the survival of patients, i.e., whether the presence of the superallele improves or deteriorates the survival rate. First, all patients were divided into two groups, i.e., patients with a particular HLA-allele and patients lacking that particular HLA-allele; subsequently, the mean survival of patients was computed in each group. Further, an allele was assigned as a survival favorable allele if the mean survival of the patients with this allele was significantly (*p*-value < 0.05) higher than the mean survival of patients without this allele. Similarly, an allele is assigned as an unfavorable allele, if the mean survival of patients with this allele is lower than the mean survival of patients without this allele. It has been observed that an individual allele is only present in a limited number of patients; thus, grouping based on the occurrence of alleles will be skewed. Therefore, we analyzed the presence and absence of HLA superalleles in patients and assigned them to survival favorable (SF) and survival unfavorable (SU) superallele groups. Here, we applied a two-sample *t*-test to check the statistical significance (*p*-value < 0.05) of these superalleles. Notably, we considered only those superallele, that must be present in at least 10 samples before assigning it to any of these groups. Further, to study the overall impact of the presence of SF and SU superalleles, we combined SF and SU superalleles and prepared a matrix; where, we assigned a score of +1 if an unfavorable superallele was present, and a score of −1 if a favorable superallele was present in an SKCM patient, otherwise 0. Eventually, all the scores were cumulatively added to generate a single score called risk score (RS). Subsequently, threshold-based methods have been developed using these superalleles as features. Finally, we assigned a patient as high-risk if the score was more than the threshold of RS, otherwise the patient was classified as low-risk.

### Survival Analysis

In the current study, “univariate” and “multivariate” survival analyses were performed by cox proportional hazard (Cox PH) models and implemented by the ‘survival’ package in R (V.3.5.1). Univariate analysis was performed to understand the impact of each variable like age, tumor stage, tumor status, gender, class I, II HLA alleles, HLA superalleles, and RS in the prognosis of cutaneous melanoma patients. Further, multivariate survival analysis was performed to understand the independent clinical impact of these HLA superalleles in the presence of other multiple factors such as age, tumor stage, tumor status, gender, and class I, II HLA superalleles (Bradburn et al., 2003). The log-rank test was used to estimate the significant survival distributions between high-risk and low-risk groups in terms of the *p*-value. Kaplan-Meier (KM) survival curves were used for the graphical representation of high-risk and low-risk groups (Kishore et al., 2010).

### Development of Prediction Models

#### Models Based on Machine Learning Techniques

In the current study, various machine learning techniques were implemented to develop regression models for OS time prediction in cutaneous melanoma patients. These machine learning techniques include a random forest (RF), ridge, lasso, and a decision tree (DT). These techniques were implemented using python-library scikit-learn (Pedregosa et al., 2011). Regression using the decision tree method, results in the supervised machine learning model, which predicts the response variable by learning the decision rules from the predictor variables. It is a tree-based approach in which a decision tree is constructed using the recursive partitioning approach in a top–down manner (Pedregosa et al., 2011). The random forest is a supervised machine learning method that implements ensemble learning. It proceeds by assembling a number of decision trees at the time of training of a model and predicts the response variable as the average prediction of the individual trees (Geurts et al., 2006). Least absolute shrinkage and selection operator or LASSO, is a type of a linear regression method that employs the shrinkage approach. It performs the L1 regularization, which leads to the model with coefficients for predictor variables, which aids in the prediction of the response variable. On the other hand, ridge regression performs the L2 regularization to calculate the coefficients (Friedman et al., 2010). To develop prediction models, we used a wide range of features that include HLA superalleles, and clinical and demographic characteristics of the patients like age, gender, stage, tumor status, Breslow depth, and their combination.

#### Wrapper Based Feature Selection Method

Here, a recursive feature selection model was developed by adding HLA superalleles to the clinical and demographic features one-by-one. Then, survival time was predicted and followed by the computation of the hazard ratio (HR) for each combination. Briefly, every time input matrix was updated by adding a new column with a HLA superallele, which had the HR just higher than that of the previous input matrix. We repeated this process until there was no further improvement in the HR. Finally, we were left with the matrix which attained the highest HR. Subsequently, this matrix was used to build the final prediction model for the estimation of OS time.

### Evaluation of Models

#### Five-Fold Cross-Validation

In order to avoid the over-optimization in the training of models, we used standard 5-fold cross-validation (Patiyal et al., 2019). In brief, all instances are randomly divided into five sets; where, four sets are used for the training and the remaining fifth set for testing. This process is repeated five times so that each set is used for testing at least once. The final performance is calculated by averaging the performance on all five sets.

#### Parameters for Measuring Performance

The major challenge in these types of studies is to use appropriate parameters to evaluate the performance of models. In this study, we used the standard parameter HR to measure the performance of the models. HR is a measure of the effect of an intervention on an outcome of interest over time. Our regression models segregate patients into high-risk and low-risk groups by taking a median cut-off. In order to evaluate our model, we compute HR from the predicted OS time for the group of patients (high-risk or low-risk patients). Additionally, we also measured the confidence interval (CI) with HR and reported the HR at 95% CI. In order to measure the significance of prediction, we also calculated the *p*-value using the log-rank test. These parameters were implemented previously in similar kinds of studies (Schemper, 1993; Chen et al., 2012).

## Results

### Distribution of HLA Alleles

We extracted a total of 4,711 HLA alleles from 415 TCGA-SKCM patients by performing the HLA typing using xHLA software (Xie et al., 2017), out of which 367 HLA alleles were unique. Among them, 367 alleles, 237 belong to the HLA class I gene (-A,-B,-C), and 130 alleles correspond to class II genes (-DPB1, -DQB1, -DRB1). We computed the frequency distribution of different alleles in patients. Due to heterogeneity in HLA genes, all alleles were not found all individuals, so the frequency of alleles vary from patient to patient (Williams, 2001). Out of 415 patients, only 357 patients had all the six alleles of the HLA class I genes. In the case of HLA class II gene, only 264 patients had all six alleles. The complete frequency distribution of class I and class II HLA alleles in the TCGA-SKCM patients is provided in Supplementary Table S3. Among them, the most abundant (present in more than 20% of the population) class-I and class-II HLA alleles include HLA-A^∗^02:01, HLA-A^∗^01:01, HLA-C^∗^07:02, HLA-C^∗^07:01, HLA-B^∗^07:02, HLA-A^∗^03:01, HLA-DPB1^∗^04:01, HLA-DQB1^∗^03:01, HLA-DQB1^∗^02:01, HLA-DPB1^∗^02:01, HLA-DRB1^∗^07:01, HLA-DQB1^∗^05:01, HLA-DRB1^∗^15:01, respectively, as shown in Supplementary Table S3.

### Categorization of Superalleles Into Favorable and Unfavorable Groups

To understand whether an allele is favorable for the survival of the patient or not, we computed the difference in mean overall survival (MOS) of patients. HLA allele is assigned as favorable if the difference in MOS is positive, otherwise it is classified as unfavorable. For instance, the class I allele HLA-A^∗^01:01 was present in 110 patients with a MOS of 72.21 months; while MOS was reduced to 55.25 months in 291 patients that lack the class I allele. Therefore, we conclude that, HLA-A^∗^01:01 is a favorable allele as its presence enhances the MOS. Similarly, the class I allele HLA-A^∗^24:02 is present in 72 patients with a MOS of 45.73 months, and it is absent in 329 patients with a MOS of 63 months. This is an unfavorable allele as its presence decreases the MOS of patients, as represented in Supplementary Table S3. These alleles can be used to predict the risk of survival; unfortunately, this statistic could be biased as the number of patients with a particular allele is very small for most of the alleles. This prompted us to create the HLA superalleles (low-resolution HLA alleles) from the high-resolution HLA alleles on the basis of field1 (F1). Here, 367 alleles were further categorized into 121 superalleles. Out of 121 superalleles, 60 and 61 belong to class I and II, respectively. HLA-A^∗^01/02, HLA-B^∗^07, HLA-C^∗^07, HLA-B^∗^44, HLA-DPB1^∗^04/02, HLA-DQB1^∗^02/03/06/05, HLA-DRB1^∗^07/15 are the most frequent class I and class II HLA superalleles in TCGA-SKCM patients, as shown in Supplementary Figure S1. Distribution of superalleles which are present in at least ten patients, is shown in Supplementary Figure S2. The abundance of all superalleles is given in Supplementary Table S4. Further, the HLA superalleles are categorized into two groups, i.e., SF and SU, on the basis of the difference in MOS between patients with a specific HLA superallele and patients without that specific HLA superallele. Among the 24 superalleles, 9 were SF (HLA-B^∗^55, HLA-DPB1^∗^01, HLA-DPB1^∗^10, HLA-B^∗^08, HLA-B^∗^49, HLA-A^∗^01, HLA-DRB1^∗^03, HLA-C^∗^05, HLA-C^∗^07) and 15 were SU (HLA-B^∗^14, HLA-A^∗^24, HLA-DPB1^∗^05, HLA-A^∗^31, HLA-DPB1^∗^11, HLA-DRB1^∗^07, HLA-DPB1^∗^06, HLA-C^∗^14, HLA-B^∗^18, HLA-C^∗^01, HLA-B^∗^13, HLA-A^∗^30, HLA-DRB1^∗^16, HLA-B^∗^50, HLA-DRB1^∗^12) with their MOS and frequency represented in Table 1.

**TABLE 1:** Classification of HLA-superalleles in to SF and SU on the basis of mean OS difference.

HLA superalleles	#No. of Samples		#Mean OS		Mean Diff OS (P-A)	*P*-value	Risk status
		
	Present (P)	Absent (A)	Present (P)	Absent (A)			
HLA-B*55	16	385	94.58	58.46	36.12	0.002	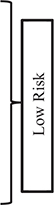
HLA-DPB1*01	34	367	87.51	57.34	30.17	6.82E-07		
HLA-B*08	80	321	81.09	54.62	26.47	6.36E-14		
HLA-DRB1*03	85	316	80.14	54.46	25.69	2.29E-14		
HLA-B*49	11	390	77.87	59.39	18.48	0.037		
HLA-A*01	115	286	72.88	54.68	18.2	1.24E-17		
HLA-C*05	61	340	72.74	57.6	15.15	1.82E-12		
HLA-DPB1*10	16	385	72.87	59.36	13.51	0.0004		
HLA-C*07	217	184	66.01	52.7	13.31	3.65E-31		
						
HLA-B*14	27	374	48.34	60.74	–12.39	2.20E-05	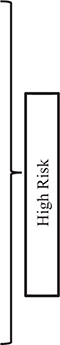
HLA-A*24	81	320	48.59	62.77	–14.18	5.61E-13		
HLA-DPB1*05	17	384	46.26	60.51	–14.25	0.001		
HLA-A*31	26	375	46.34	60.84	–14.5	1.76E-05		
HLA-DPB1*11	10	391	45.32	60.27	–14.95	0.003		
HLA-DRB1*07	103	298	48.37	63.89	–15.51	4.31E-14		
HLA-DPB1*06	12	389	43.68	60.4	–16.72	0.014		
HLA-C*14	10	391	43.44	60.32	–16.88	0.003		
HLA-B*18	39	362	44.41	61.57	–17.16	1.07E-08		
HLA-C*01	42	359	44.35	61.72	–17.37	9.08E-07		
HLA-B*13	19	382	41.94	60.79	–18.86	0.03		
HLA-A*30	26	375	42.14	61.13	–19	5.22E-06		
HLA-DRB1*16	23	378	29.53	61.75	–32.22	7.00E-06		
HLA-B*50	12	389	25.03	60.98	–35.95	6.33E-05		
HLA-DRB1*12	19	382	23.46	61.71	–38.26	9.43E-05		
						

*#Samples (P): No of SKCM-patients in which HLA-superallele is present; # Samples (A): No of SKCM-patients in which HLA-superallele is absent; #Mean OS (P): Average OS in which HLA-superallele is present; # Mean OS (A): Average OS in which HLA-superallele is absent; Mean Diff OS: Mean difference in mean OS between patients with a specific HLA typing and patients without it; Risk status Survival Favorable (SF) or Survival Unfavorable (SU) HLA-superallele, SF considered as low-risk and SU taken as high-risk groups.*

### Univariate Survival Analysis

#### HLA Superalleles

It is clear from the above analysis that certain alleles/superalleles are essential for the survival of cutaneous melanoma patients. The next challenge is to utilize this information to predict high-risk cancer patients based on the presence of key alleles or superalleles. Here, we used HLA superalleles to predict high-risk patients, employing the univariate survival analysis due to the poor distribution of alleles in patients. We observed that HLA-B^∗^50, which is responsible for poor survival in patients, assigns patients as a high risk if this superallele is present and obtained a HR of 2.77 (95% CI 1.284 to 5.941) with a *p*-value of 0.009. Similarly, HLA-DRB1^∗^12 achieved the maximum performance of HR 3.13 (95% CI 1.687–5.826) with a *p*-value < 0.001. The combined effect of the presence of HLA-B^∗^50 and HLA-DRB1^∗^12 was also used to predict high-risk patients and obtained HR 3.15, 95% (CI 1.906–5.194) with a *p*-value less than 0.001, as shown in Supplementary Table S5.

#### Risk Score (RS)

**FIGURE 3:**
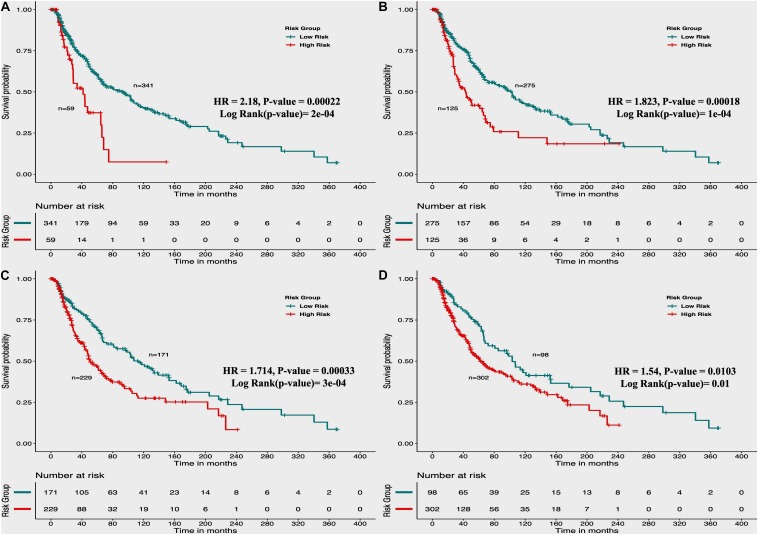
Kaplan Meier (KM) survival curves for the risk estimation of melanoma patient cohort based on the risk score with significant *p*-value **(A)** Melanoma samples stratified on the basis of cut-off (≥2 Risk Score), **(B)** Stratified samples by taking cut-off (≥1 Risk Score), **(C)** Stratified samples by taking cut-off (≥0 Risk Score), **(D)** Stratified samples by taking cut-off (≥–1 Risk Score).

From the above univariate analysis, we have identified key HLA superalleles, which exhibit a significant role in the prognosis of melanoma patients as a single feature. We next aimed to use them as features for the development of prediction methods. Therefore, we developed a threshold-based method using RS, which was derived from multiple HLA superalleles. To understand how well RS based on multiple superalleles stratified risk-groups of cutaneous melanoma patients, a survival analysis was performed using this RS as an input feature. For instance, if the threshold value is ≥2, then the patients are significantly divided into high-risk and low-risk groups with HR 2.18 (95% CI 1.441–3.297) and a *p*-value of 0.000223, as given in Table 2. Conclusively, we found that RS thresholds act as a prognostic indicator to stratify melanoma patients into high-risk and low-risk groups, as shown in Table 2. Additionally, KM survival plots represent the segregation of risk groups of melanoma patients based on different threshold values of RS (shown in Figure 3).

**TABLE 2:** Survival analysis based on Risk score to discriminate low-risk and high-risk samples.

Threshold (Risk Score)	#G1	#G2	HR	95% CI	*P*-value
≥3	375	26	1.84	0.97–3.51	6.35E-02
≥2	341	60	2.18	1.44–3.30	2.23E-04
≥1	275	126	1.82	1.33–2.50	1.83E-04
≥0	171	230	1.71	1.28–2.30	3.35E-04
≥−1	98	303	1.55	1.11–2.16	1.03E-02
≥−2	61	340	1.26	0.87–1.82	0.23
≥−3	37	364	1.55	0.98–2.46	0.06

*#G1: No of SKCM-patients representing low-risk group; #G2: No of SKCM-patients denoting high-risk group; HR: Hazard Ratio; 95% CI: 95% confidence interval.*

#### Clinical and Demographic Characteristics

**FIGURE 4:**
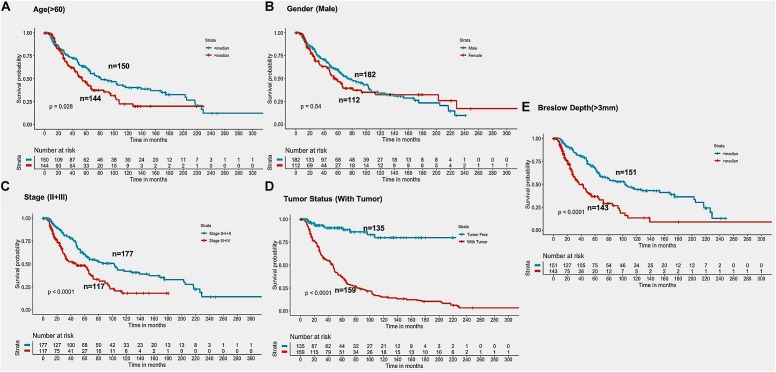
Kaplan Meier survival curves for risk estimation of SKCM cohort, show a significant difference in the high-risk/low-risk groups. **(A)** Patients with age (>60 years) are stratified into high/low risk with HR = 1.45, 95%CI = 1.039–2.024 and *p*-value = 0.028, **(B)** Stratification of low-risk and high-risk groups on the basis of gender with HR = 1.11, 95%CI = 0.7901–1.52, and *p*-value = 0.545, **(C)** Stage (III + IV) patients are on high risk as compared to Stage (0 + I + II) patients with HR = 1.94, 95%CI = 1.386-2.722, *p*-value < 0.001, **(D)** Patients with Tumor status (With Tumor) were stratified on high/low-risk with HR = 8.29, 95%CI = 4.688–14.67, and *p*-value < 0.001, **(E)** Patients having Breslow depth > 3 mm are stratified into high/low-risk corresponding 95%CI 1.788–3.509, HR = 2.5, and *p*-value < 0.001.

In the past, clinical and demographic features like age, gender, tumor stage, tumor status, and Breslow depth, have shown a significant effect on skin cancer incidence and a bias toward a particular group (Zhang and Zhang, 2017). For instance, even in the current study, male incidences are higher than that of females, as shown in Supplementary Table S1. This prompted us to analyze the association between these clinical features and the survival of patients. Thus, we performed a univariate survival analysis using these clinical and demographic features. This analysis indicates that the tumor status is a major significant prognostic factor in the prediction of survival time of melanoma patients. We predict patients to be at high-risk if the score is more than zero and obtained HR 8.293 (95% CI 4.688–14.67) with a *p*-value of less than 0.0001 (Supplementary Table S6). Age, tumor stage, and Breslow depth are other features that are significantly associated with the prognosis of patients, as shown in Figure 4. However, samples are unable to stratified into high-risk and low-risk groups based on the gender (Figure 4).

### Prediction Models

#### Machine Learning Based Prediction Models

It is clear from the above results that HLA superalleles, clinical and demographic features (such as age, gender, tumor stage, tumor status, and Breslow depth) are essential to identify high-risk patients. The threshold-based method, however, is simple, but not very efficient when multiple features were used. Thus, to further improve the performance, we implemented a wide range of machine learning techniques (such as, lasso, RF, ridge, DT) to develop prediction models. First, we considered all 121 superalleles to develop machine learning models. The RF model is the top performing model and achieves a maximum HR of 3.264, and a *p*-value of 1.03E-10, as represented in Supplementary Table S7. Subsequently, a prediction model was developed by considering clinical, demographic, and 24 HLA superalleles as input features. In addition, Lasso and RF based models were also developed using only clinical and demographic features (Feature Set-1) and obtained a maximum performance with HR 3.17 (*p*-value 3.50E-11), and HR 3.09 (*p*-value 2.87E-11), respectively, as shown in Table 3. Further, we developed models by eliminating two factors, i.e., tumor status and tumor stage. Although the tumor stage is an important clinical factor, this information is sometimes available only for a few patients. So, the prediction model was developed without considering these clinical factors and a maximum HR of 2.99 (with *p*-value 9.37E-12) was achieved by the RF model. To further improve the performance of the machine learning based models, we used all clinical and demographic features with the key 24 HLA superalleles (Feature Set-2). Models based on the lasso regressor achieved the maximum performance with a HR 4.05 and a significant *p*-value of 4.01E-13. However, RF prediction models also performed reasonably well, but had a lower HR than that of the lasso models. The complete results of the survival prediction models are represented in Table 3. It has been reported in the literature that class I alleles are important in tumor cell elimination (Garcia-Lora et al., 2003; Chang et al., 2005). Therefore, we also tried to build prediction models employing class-I alleles only. Here, the prediction model was developed using 15 class I superalleles, two demographic, and three clinical features. RF performs best among other machine learning models with a HR of 2.91 and a *p*-value of 1.79E-07. The complete results are shown in Supplementary Table S8.

**TABLE 3:** Performance of the survival prediction models based on Clinical Characteristics and 24 HLA-Class I, II Superalleles implemented using various regression techniques.

Method	Feature Set-1		Feature Set-2	
		
All Features				

	HR	*P*-value	HR	*P*-value
LASSO	3.17	3.50E-11	4.05	4.01E-13
RIDGE	3.01	1.76E-10	3.80	2.30E-12
RF	3.09	2.87E-11	3.77	8.15E-12
DT	2.25	6.93E-07	2.00	5.29E-05
**Clinical features without tumor status**				
LASSO	3.50	3.93E-13	3.46	1.54E-11
RIDGE	3.49	3.93E-13	2.97	2.89E-09
RF	3.74	3.01E-14	2.96	8.23E-10
DT	2.15	2.24E-06	1.83	3.12E-04
**Clinical features without tumor stage**				
LASSO	2.80	9.96E-10	3.51	1.32E-11
RIDGE	2.43	4.68E-08	3.55	7.56E-12
RF	2.81	2.05E-10	3.18	2.14E-10
DT	2.50	1.64E-08	2.76	2.38E-09
**Clinical features without tumor stage and tumor status**				
LASSO	2.40	4.41E-08	3.11	5.60E-10
RIDGE	2.40	4.41E-08	2.57	5.81E-08
RF	2.99	9.37E-12	2.59	1.55E-08
DT	2.54	1.06E-08	2.65	7.37E-09

*#HR: Hazard Ratio; RF: Random Forest; DT: Decision Tree; Feature Set-1: Clinical and demographic features, Feature Set-2: Clinical, demographic and 24 HLA superalleles features.*

#### Machine Learning Prediction Models Based on Wrapper Method

**FIGURE 5:**
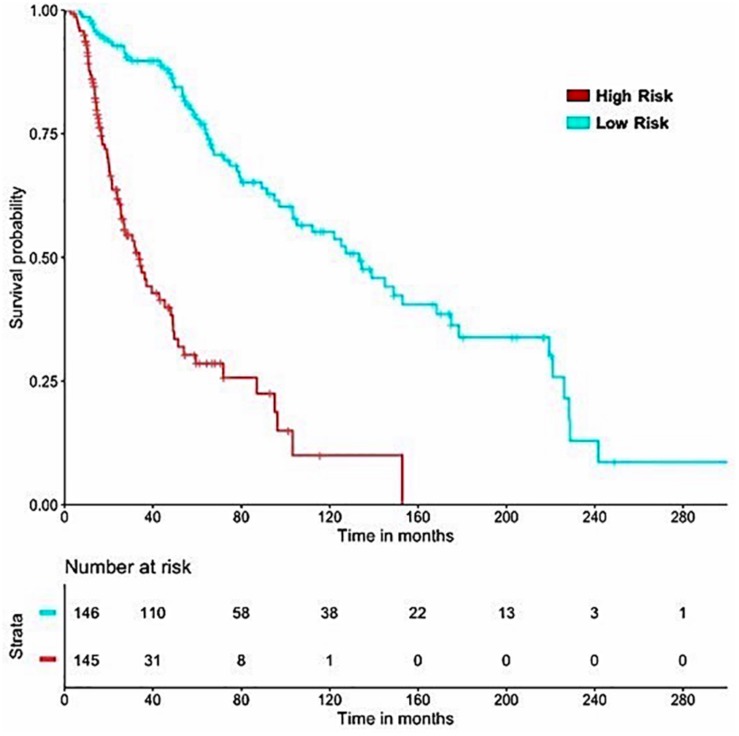
SKCM-patients were stratified based on predicted OS by using Lasso recursive regression model after applying fivefold cross validation. Samples with predicted OS < median (predicted OS) were at fourfold higher risk as compared to the patients predicted OS > median (predicted OS) (HR = 4.52, 95% CI = 3.088 to 6.609, *p*-value = 8.01E-15).

It is important to have a minimum number of features to avoid over-optimization and for practical implementation in real life. Therefore, a further wrapper method was used to decrease the number of features recursively. Finally, prediction models were developed using five clinical and demographic characteristics (age, gender, tumor stage, tumor status, and Breslow depth) and various HLA superalleles, by implementing different machine learning techniques. Similar to the above analysis, the lasso method, based on five clinical features and 14 superalleles, was the top performer with a HR of 4.52 and a *p*-value of 8.01E-15, as given in Table 4. The KM plot represents the stratification of high-risk and low-risk patients based on the estimated OS using the lasso recursive regression model, as shown in Figure 5.

**TABLE 4:** Performance of the recursive prediction models based on selected features (clinical features and superalleles) implemented using various regression technique.

Method	Attribute	HR	*P*-value
LASSO	Clinical + 14 HLA-superalleles (A*31_A*24_DPB1*10_B*08_DRB1* 03_DRB1*07_B*18_B*55_A*01_C* 05_DRB1*16_DRB1*12_B*49 _DPB1*11)	4.52	8.01E-15
RIDGE	Clinical + 19 HLA-superalleles (DPB1*10_B*50_C*07_B*49_B*55 _B*08_C*01_C*14_DPB1*06_C*05 _DRB1*03_A*30_DRB1*07_A*31 _B*14_DRB1*16_B*13_DPB1* 01_A*01)	3.85	3.35E-12
RF	Clinical + 3 HLA-superalleles (DPB1*11_C*05_B*08)	3.53	2.84E-11
DT	Clinical + 2 HLA-Superalleles (A*01_DPB1*01)	2.59	6.92E-08

*#HR: Hazard Ratio; RF: Random Forest; DT: Decision Tree; Attribute: Clinical features and selected HLA-superalleles.*

#### Performance on the External Validation Dataset

In order to evaluate performance on the external validation dataset, we considered only 27 available input features, which included 24 superalleles, two demographic (age and gender), and one clinical feature (tumor stage) for both training and validation, represented as ‘Set-A.’ Here, training was done on the TCGA-SKCM dataset and validated on the external dataset. The prediction model based on lasso, with 24 HLA superalleles, stratified the risk groups with a HR of 2.66 (*p*-value 6.94E-08) and 2.24 (*p*-value 0.000778) for training and validation datasets, respectively. Whereas, after applying the wrapper method we got 14 HLA superalleles and then, we trained and validated our model on these 14 HLA superalleles along with two demographic and one clinical feature, which resulted in 17 input features, represented as ‘Set-B.’ The performance of the lasso model using features of ‘Set-came out’ with a HR of 2.81 (*p*-value 1.06E-08) and a HR of 2.11 (*p*-value 0.0018) for the training and validation dataset, respectively, as shown in Table 5.

**TABLE 5:** Performance of the prediction models based on lasso method using selected features (2 demographic and 1 clinical features, 24 and 14 HLA superalleles) on training and external validation dataset.

Dataset	Set-A		Set-B	
		
	HR	*P*-value	HR	*P*-value
Training data (TCGA-SKCM)	2.66	6.94E-08	2.81	1.06E-08
External validation data	2.24	0.000778	2.11	0.0018

*#HR: Hazard Ratio; Set-A: 1 Clinical, 2 demographic features and selected 24 HLA-superalleles; Set-B: 1 Clinical, 2 demographic features and selected 14 HLA-superalleles.*

### Multivariate Survival Analysis for SF and SU HLA Superalleles

Further, to understand the independent impact of the different variables, like SF and SU HLA-superalleles, RS, and clinical and demographic features in the presence of all the factors, on the survival of the patients, we performed a multivariate survival analysis using the cox proportional hazard model (Bradburn et al., 2003). This analysis revealed that RS is a significant independent factor associated with the survival of patients. Results (shown in Supplementary Figure S2) indicate that the presence of SU superalleles reduces the survival of melanoma samples. The SU patients group is at approximately two times higher risk as compared to the SF patients group as indicated by a HR of 2.44 (95% CI 1.68–3.5) with a p-value less than 3.02E-06 (shown in Table 6). Both multivariate and univariate analysis revealed that age (>60), stage (III and IV), Breslow depth (>3 mm), and RS (>0) are associated with poor survival in melanoma patients, as represented in Supplementary Figure S3.

**TABLE 6:** Comparison of univariate and multivariate analysis.

	Univariate survival analysis			Multivariate survival analysis		
		
Covariate	HR	95% CI	*P*-value	HR	95% CI	*P*-value
Age (>60 years)	1.45	1.04–2.02	0.029	1.45	1.03–2.00	3.20E-02
Gender (Female)	1.11	0.79–1.52	0.545	0.98	0.69–1.40	0.896
Tumor Stage (III + IV)	1.94	1.39–2.72	0.001	1.89	1.33–2.70	4.00E-04
Tumor Status (With Tumor)	8.29	4.69–14.67	<0.001	9.24	5.21–2.80	2.76E-14
Breslow Depth(>3 mm)	2.50	1.79–3.51	<0.001	1.96	1.38–2.80	1.70E-04
Risk Score (>0)	1.82	1.33–2.50	<0.001	2.44	1.68–3.50	3.02E-06

*HR: Hazard Ratio, 95% CI: 95% Confidence Interval.*

Further, to scrutinize which specific superalleles out of SF and SU superallele groups, are significantly associated with good and poor outcomes in patients, multivariate analysis was performed using each of the SF and SU superalleles with the clinical and demographic characteristics. Results from this analysis show that the presence of HLA-B^∗^55 and HLA-A^∗^01 superalleles is significantly associated with a good outcome; while, HLA-DRB1^∗^12, HLA-B^∗^50, HLA-B^∗^13, HLA-DPB1^∗^06, HLA-A^∗^31, HLA-A^∗^24 is significantly associated with a poor outcome of a melanoma cohort in terms of their survival time, as given in Supplementary Tables S9, S10 and Supplementary Figures S4, S5.

### Web Server for Risk Prediction in SKCM Patients: SKCMhrp

To serve the scientific community, we developed a web server, “SKCMhrp” https://webs.iiitd.edu.in/raghava/skcmhrp/. SKCMhrp is designed to predict risk using clinical, demographic features and HLA superalleles. It has two modules; one is based on clinical features and the second is based on superalleles. The first module predicts the risk status of melanoma patients based on their clinical and demographic characteristics, i.e., age, gender, tumor stage, tumor status, Breslow depth. Here, a user can predict the survival time (in months) of the individual sample, even by choosing a single clinical feature. Input values are given to a regression model to estimate the risk status. The second module predicts the risk status of melanoma patients using all 121 superalleles and 14 superalleles with five clinical and demographic features. The webserver “SKCMhrp” was built using HTML, PHP 5.2.9, and JAVA scripts. To make the website compatible with mobiles and tablets, we used the HTML5 web template. The abovementioned technologies that have been implemented are open source and platform-independent.

## Discussion

Skin cutaneous melanoma is a lethal malignancy as indicated by the rise in incidence of melanoma (Siegel et al., 2020). The FDA (Food and Drug Administration) has approved several therapies and strategies to curb melanoma in the past few years. Choosing a treatment from the available options, however, requires information about the tumor such as its location, stage, etc. Accurate tumor stage identification with high precision is itself a challenging task in different malignancies (Jagga and Gupta, 2014; Bhalla et al., 2017, 2018; Saghapour et al., 2017; Kaur et al., 2019; Yu et al., 2019). Recent findings have suggested that antigenic repertoire variability is a crucial factor in tumor progression and immunosurveillance (Dunn et al., 2004). For instance, HLA-class I and II proteins have been shown to have a significant role in the progression of melanoma (Gogas et al., 2010; Bonamigo et al., 2012; Kandilarova et al., 2016). Thus, it is important to understand which specific HLA alleles from class-I and class-II could affect the survival of the patients. To this end, the current study is a systematic attempt to understand the prognostic roles of class-I/II alleles in the survival of melanoma patients.

In this study, 367 unique HLA alleles were identified for 415 cutaneous melanoma patients using the xHLA software. The low-frequency distribution of these 367 alleles among patients (as shown in Supplementary Table S3) made it difficult to delineate any reliable conclusion regarding any of the alleles from the analysis. This propelled us for their assignment into low-resolution HLA typing, i.e., 121 HLA superalleles. Thereafter, these superalleles were categorized into SF and SU groups based on the impact of their presence on the survival of the patients, i.e., higher MOS or lower MOS of the patients with their occurrence, respectively. Here, among the 24 superalleles, nine were SF including HLA-B^∗^55, HLA-DPB1^∗^01, HLA-DPB1^∗^10, HLA-B^∗^08, HLA-B^∗^49, HLA-A^∗^01, HLA-DRB1^∗^03, HLA-C^∗^05, HLA-C^∗^07; while, 15 were SU that include HLA-B^∗^14, HLA-A^∗^24, HLA-DPB1^∗^05, HLA-A^∗^31, HLA-DPB1^∗^11, HLA-DRB1^∗^07, HLA-DPB1^∗^06, HLA-C^∗^14, HLA-B^∗^18, HLA-C^∗^01, HLA-B^∗^13, HLA-A^∗^30, HLA-DRB1^∗^16, HLA-B^∗^50, HLA-DRB1^∗^12. In the literature, HLA-A^∗^01, HLA-C^∗^05, and HLA-C^∗^07 have been shown to be positively associated with survival of melanoma patients (Paschen et al., 2005; Campillo et al., 2006; Zhu et al., 2012), whereas HLA-B^∗^14, HLA-A^∗^24, HLA-A^∗^31, HLA-C^∗^14, and HLA-B^∗^13 are negatively associated with survival of melanoma patients (Marincola et al., 1995; Kawakami et al., 2000; Akiyama et al., 2005; Kandilarova et al., 2016; Rogel et al., 2019). Apart from these, HLA-DRB1^∗^07 has been shown to be negatively associated with patient survival in other cancers such as lung cancer, cervical cancer, and breast cancer (Ferreiro-Iglesias et al., 2018; Hu et al., 2018; Spraggs et al., 2018). Further, in the current study, a parameter RS was computed to evaluate the cumulative effect of the presence of SF and SU superalleles in patients. After that, 24 HLA superalleles, and clinical features like tumor status, Breslow depth, and tumor stage were identified which can significantly stratify high-risk and low-risk survival groups, by employing univariate survival analysis and a log rank test (Supplementary Tables S5, S6). Furthermore, prediction models were developed based these 24 superalleles, and clinical and demographic features, which stratified the risk groups with HR 4.05 (*p*-value 4.01E-13). In the past, the role of class I alleles was reported to be crucial for the defense against a tumor. Therefore, prediction models were developed by employing 15 class I superalleles with clinical and demographic features only. The RF model attained the maximum HR of 2.9 (*p*-value 1.79E-07). This indicates that not only class I, but rather both class I and II superalleles are important in the stratification of survival risk groups as performance decreases on the exclusion of class-II superalleles. Subsequently, the performance of the model based on 24 superalleles with age, gender, and tumor stage was also evaluated on the external validation dataset. This model stratified survival risk groups of the external dataset with a HR of 2.24 (*p*-value 0.000778). Recently, Chen et al. (2019) also reported that the higher expression of HLA-class II genes enhances the survival of melanoma patients. Our analysis also revealed that, the higher expression of HLA (-A, -B, -C, -DPB1, -DQB1, -DRB1) genes, are associated with poor survival, as shown in Supplementary Table S11. Besides, our study also indicates that stage is a major prognostic factor for melanoma patients. It significantly stratified high-risk and low-risk patients in both univariate and multivariate analyses with a *p*-value of 0.001 and 4.00E-04, respectively (Table 6). It corroborates with previous literature as well; where, it has been reported that the tumor stage of melanoma patients drastically affects their prognosis. For instance, the OS of stage-Ia patients is quite good, i.e., a 10-year survival of 95%, while it is only 30% in the case of stage-IV patients. A moderate survival rate has been reported in the case of stage 1b, II, and III patients, which is shown to vary between 85 and 40% (Balch et al., 2009; Yélamos and Gerami, 2015).

Multivariate survival analysis was performed to better understand the prognostic impact of the association of SF and SU superalleles with the clinical and demographic features on the survival of patients. This analysis revealed that SF and SU superalleles also act as independent prognostic indicators. For instance, the presence of HLA-class I superalleles, such as HLA-B^∗^55 (HR = 0.15, *p*-value = 0.013) and HLA-A^∗^01(HR = 0.54, *p*-value = 0.011) is significantly associated with the good outcome (Supplementary Table S9 and Supplementary Figure S4). On the other hand, the presence of superalleles such as HLA-B^∗^50 (HR = 3.1 and *p*-value = 0.03), HLA-DRB1^∗^12 (HR = 3.77 and *p*-value < 0.001), HLA-DRB1^∗^16 (HR = 2.18, *p*-value = 0.04), HLA-B^∗^13 (HR = 2.49, *p*-value = 0.046), HLA-DPB1^∗^06 (HR = 3.53, *p*-value = 0.006), HLA-A^∗^31 (HR = 2.09, *p*-value = 0.04), and HLA-A^∗^24 (HR = 1.79, *p*-value = 0.006) is associated with a poor survival outcome (Supplementary Table S10 and Supplementary Figure S5). In addition to this, RS, tumor status, tumor stage, Breslow depth, and age were also revealed as major independent prognostic factors for melanoma patients.

Furthermore, with an aim to estimate the survival time of melanoma patients, various regression models were developed based on survival-associated superalleles, clinical and demographic features, and their combination. For this, we implemented diverse machine learning regressors like lasso, RF, DT, and ridge regressor. The predicted OS from these models was further employed for the stratification of high-risk and low-risk survival groups. Although the prediction, based on five clinical and demographic factors, attained a consistent performance (HR = 3.17). The accurate determination of the stage and tumor status remains a difficult task. Therefore, prediction models were also developed after the exclusion of these two factors. The performance of our ML models substantially decreased to a HR of 2.99. Thereafter, prediction models were developed based on HLA-superalleles and conveniently available demographic and clinical factors like age, gender, and Breslow depth. The performance improved considerably from a HR of 2.99 to 3.11. Lasso and RF recursive regression models were found to be among the top performers for the prediction of the survival of melanoma samples. In particular, predicted OS obtained from the lasso recursive model, based on clinical and demographic characteristics and 14 superalleles, significantly (*p*-value = 8.01E-15) stratified the high-risk and low-risk survival groups of the cutaneous melanoma patients with a HR = 4.52. Although the RF-based models performed reasonably well in the estimation of OS, however, they achieved a lower HR of 3.53 than that of lasso models. The performance of this model was further evaluated on an external validation dataset considering 14 superalleles with age, gender, and tumor stage; attained an HR 2.11 (*p*-value 0.0018), as represented in Table 5.

## Conclusion

Altogether, our findings show that the presence of HLA-class I and II alleles influence the OS of TCGA-SKCM patients both favorably and unfavorably. Eventually, survival analysis and recursive machine learning regression models revealed the prognostic potential of 14 superalleles, clinical and demographic features in the stratification of high-risk and low-risk survival groups and the estimation of OS time. Further, these HLA-based signatures could be considered in the design of personalized vaccines in several clinical cohorts. For clinical utility, this needs to be further confirmed by exploring the role of these superalleles in other cohorts. Finally, to provide a service to the scientific community to predict high-risk patients based on their clinical features, demographic features, 14 and 121 HLA-superalleles, we designed a webserver “SKCMhrp.”

## Limitation of the Study

In the current study, the prognostic potential of 14 superalleles (low-resolution HLA allele), demographic and clinical features were revealed to estimate the survival of cutaneous melanoma patients. One of the limitations of these features/markers is that they are derived from low-resolution HLA alleles. Researchers can, however, implement a similar strategy if sufficient data is available for high-resolution HLA alleles. Further, we have not considered the ethnicity of the patients for the analysis or development of the prediction models in this study.

## Data Availability Statement

All the datasets generated for this study are either included in this article/Supplementary Material or available at the “SKCMhrp” webserver https://webs.iiitd.edu.in/raghava/skcmhrp/data.php, as mentioned in the “Materials and Methods” section.

## Author Contributions

AD, HK, and SB collected and processed the datasets. AD, HK, and SP implemented the algorithms. AD and SP created the back-end of the web server and front-end user interface. AD and SP developed the prediction models. AD, HK, and GR analyzed the results. AD, HK, SP, CA, and GR penned the manuscript. GR conceived and coordinated the project and provided overall supervision to the project. All authors have read and approved the final manuscript.

## Conflict of Interest

The authors declare that the research was conducted in the absence of any commercial or financial relationships that could be construed as a potential conflict of interest.
